# Similar and different? A cross-cultural comparison of the prevalence, course of and factors associated with suicidal thoughts and behaviors in first-episode psychosis in Chennai, India and Montreal, Canada

**DOI:** 10.1177/00207640231214979

**Published:** 2024-01-04

**Authors:** Roxanne Sicotte, Amal Abdel-Baki, Greeshma Mohan, Daniel Rabouin, Ashok Malla, Ramachandran Padmavati, Laura Moro, Ridha Joober, Thara Rangaswamy, Srividya N. Iyer

**Affiliations:** 1 Research Center of the Centre Hospitalier de l’Université de Montréal (CRCHUM), Montréal, QC, Canada; 2 Department of Psychiatry and Addiction, Faculty of Medicine, Université de Montréal, Montréal, QC, Canada; 3 Schizophrenia Research Foundation (SCARF), Chennai, Tamil Nadu, India; 4 Prevention and Early Intervention Program for Psychosis (PEPP), Douglas Mental Health University Institute, Montréal, QC, Canada; 5 Department of Psychiatry, Faculty of Medicine and Health Sciences, McGill University, Montréal, QC, Canada; 6 Department of Psychology, Faculty of Arts and Sciences, Université de Montréal, Montréal, QC, Canada

**Keywords:** First-episode psychosis, early intervention services, suicidal ideation, suicide attempts, low- and middle-income countries, high-income countries

## Abstract

**Background:**

Data from high-income countries (HICs) show a high risk of suicidal thoughts and behaviors (STBs) in first-episode psychosis (FEP). It is unknown, however, whether rates and associated factors differ in low- and middle-income countries (LMICs).

**Aims:**

We therefore aimed to compare the 2-year course of STBs and associated factors in persons with FEP treated in two similarly structured early intervention services in Chennai, India and Montreal, Canada.

**Method:**

To ensure fit to the data that included persons without STBs and with varying STBs’ severity, a hurdle model was conducted by site, including known predictors of STBs. The 2-year evolution of STBs was compared by site with mixed-effects ordered logistic regression.

**Results:**

The study included 333 FEP patients (168 in Chennai, 165 in Montreal). A significant decrease in STBs was observed at both sites (OR = 0.87; 95% CI [0.84, 0.90]), with the greatest decline in the first 2 months of follow-up. Although three Chennai women died by suicide in the first 4 months (none in Montreal), Chennai patients had a lower risk of STBs over follow-up (OR = 0.44; 95% CI [0.23, 0.81]). Some factors (depression, history of suicide attempts) were consistently associated with STBs across contexts, while others (gender, history of suicidal ideation, relationship status) were associated at only one of the two sites.

**Conclusions:**

This is the first study to compare STBs in FEP between two distinct geo-sociocultural contexts (an HIC and an LMIC). At both sites, STBs reduced after treatment initiation, suggesting that early intervention reduces STBs *across* contexts. At both sites, for some patients, STBs persisted or first appeared during follow-up, indicating need for suicide prevention throughout follow-up. Our study demonstrates contextual variations in rates and factors associated with STBs. This has implications for tailoring suicide prevention and makes the case for more research on STBs in FEP in diverse contexts.

## Introduction

Worldwide, suicide causes 703,000 deaths annually. Suicide rates are higher in high-income countries (HICs; 10.9/100,000) than in lower-middle-income (10.1/100,000) and low-and upper-middle income countries (9.9/100,000; World Health Organization [WHO], 2021). Nonetheless, most (77%) suicides occur in low- and middle-income countries (LMICs), where the majority of the world’s population lives (WHO, 2021). In HICs, middle-aged men generally have higher suicide rates than in many LMICs, where, young adults and older women have higher suicide rates (WHO, 2014, 2021). While over 90% of persons deceased by suicide are estimated to have a mental disorder in HICs (Arsenault-Lapierre et al., 2004), psychiatric disorders may play a lesser role in LMICs, with other risk factors being more important (e.g. family, intergenerational and interpersonal conflicts/difficulties, economic stressors/poverty)(Knipe et al., 2019; Liu et al., 2018; Rane & Nadkarni, 2014; Uddin et al., 2019).

Suicidal thoughts and behaviors (STBs; suicide ideation, plans, and attempts) are major risk factors for suicide (Franklin et al., 2017; Turecki et al., 2019), which is a leading cause of death in persons with psychotic disorders (Kurdyak et al., 2021), with suicide death rates ranging from 5% to 9% (Knipe et al., 2019). Meta-analyses have reported higher prevalence of STBs in schizophrenia in HICs than in LMICs (Bai et al., 2021; Lu et al., 2020); but these meta-analyses were not about first-episode psychosis (FEP). Multiple HIC studies reported a particularly high risk of death by suicide and STBs in FEP compared to later illness stages (Nordentoft et al., 2004; Simon et al., 2018; Zaheer et al., 2020). In HICs, 30.6% to 56.5% of FEP patients had suicidal ideation when they first entered services and 3.3% to 9.6% attempted suicide (Sicotte et al., 2021).

Rates of STBs in FEP have rarely been studied in LMICs (Sicotte et al., 2021). Psychosis outcomes (symptoms, disability, functioning, engagement) have been found to be more favorable in some LMICs (Harrison et al., 2001; Hopper & Wanderling, 2000; S. N. Iyer et al., 2022; Malla et al., 2020) compared to HICs, including in our India-Canada study of which the present report is a part (Malla et al., 2020). Questions thus remain about whether these more favorable outcomes in FEP in LMICs (Cohen et al., 2008; Sicotte et al., 2021) extend to STBs.

Factors associated with STBs can help better target patients with an increased suicidal risk. In FEP, these factors, mainly identified in HICs, include history of STBs, depressive symptoms, substance use disorder and hopelessness (Challis et al., 2013; Coentre et al., 2017; McGinty et al., 2018; Sicotte et al., 2021). Factors associated with STBs may differ across contexts (Altamura et al., 2007; Bhatia et al., 2006). A cross-site study of schizophrenia or schizoaffective disorder (*not* FEP) reported that the type of psychotic disorder, negative symptoms, depression, and low education were associated with higher risk of suicide attempts in an American cohort, whereas none of these factors were significant in an Indian cohort (Bhatia et al., 2006).

A cross-cultural understanding of STBs is essential in the early illness stage which is critical for long-term outcomes (Birchwood et al., 1998; Harrison et al., 2001) and a period of heightened risk for STBs. Cross-cultural studies are more trustworthy when confounds (e.g. nature of previous treatment) are minimized by comparing similarly defined FEP cohorts receiving similar services (S. N. Iyer et al., 2010). No study has undertaken such a comparison. Cross-cultural comparison can also help identify specific and common risk factors and inform suicide prevention strategies (Nock et al., 2008).

Addressing this gap, our objectives were to (1) compare factors associated with STBs in FEP patients treated in two similarly structured early intervention services (EIS) in an HIC (Montreal, Canada) and an LMIC (Chennai, India) and (2) compare the 2-year course of STBs across sites.

## Material and methods

### Settings and sample

This prospective study was conducted in two EIS for psychosis in Montreal, Canada and Chennai, India (Malla et al., 2020). In Montreal, the Prevention and Early Intervention Program for Psychosis (PEPP), which is part of a publicly funded healthcare system, includes a larger service in southwest Montreal serving a defined catchment (population of 300,000), and a smaller service serving central Montreal (no defined catchment). In Chennai, the Schizophrenia Research Foundation (SCARF), a mental health non-governmental organization, houses an EIS that does not serve a strictly defined catchment.

Both EIS provide similar 2-year specialized care based on international EIS guidelines (Early Psychosis Guidelines Writing Group and EPPIC National Support Program, 2016; IRIS, 2012), including open referral systems, low-dose antipsychotic medication, psychiatric follow-up, individual and family interventions, and recovery-oriented case management (S. Iyer et al., 2015). Both sites integrate adaptations to fit local context, for example, yoga and cognitive remediation focused on household chores in Chennai (S. N. Iyer et al., 2022; Rangaswamy et al., 2012).

At both sites, in cases of STBs, in-person and phone contacts with the patient and family were increased; resources (e.g. suicide helplines) provided, and psychiatric hospitalization was available if needed. Additional resources in Montreal included hospital beds dedicated to EIS, crisis centers and emergency services within the EIS’ larger premises. In Chennai, clinicians often asked families to be more involved and observant in cases of STBs.

Research ethics boards at both sites approved this study and all patients provided informed consent. All consecutive patients were approached to participate in the study upon admission. Inclusion criteria were: primary diagnosis of schizophrenia-spectrum or affective psychotic disorder according to the Structured Clinical Interview for DSM-IV (First et al., 2002), no prior treatment with antipsychotic medication for >30 days, age 16 to 35, IQ > 70, and ability to communicate in French/English in Montreal and Tamil/English in Chennai. Exclusion criteria were: psychotic disorder secondary to a general medical condition or substance-induced psychosis. Comorbid substance use disorders were not an exclusion criterion.

### Assessments

At both sites, assessments were administered by similarly and rigorously trained research staff. Well-established measures were used, high inter-rater reliability across sites was reported (Malla et al., 2020), and data management was centralized.

#### STBs

STBs were systematically assessed at seven time points (baseline, months 2, 3, 6, 12, 18, 24) using the 4-point suicide item of the Calgary Depression Scale for Schizophrenia (CDSS) (Addington et al., 1993), referring to the last month: 0 (absent), 1 (suicide ideation), 2 (suicide ideation with a plan), and 3 (suicide attempt). At both sites, deaths by suicide were recorded. In Montreal, for all persons lost to follow-up during the study period, a request was made to the coroner’s office to verify if there was a report of suicide. No patients were lost to follow-up in Chennai (Early Psychosis Guidelines Writing Group and EPPIC National Support Program, 2016).

#### Baseline clinical and demographic variables

Informed by the literature, data were collected on factors potentially associated with STBs (gender, age, relationship status, duration of untreated psychosis (DUP), positive and negative symptoms, functioning, depressive symptoms, history of STBs).

Detailed sociodemographic data were collected at baseline. DUP, defined as the time (in weeks) from the onset of the presenting episode to the initiation of continuous antipsychotic treatment (S. N. Iyer et al., 2010), was assessed with the semi-structured interview, Circumstances of Onset and Relapse Schedule (CORS; Norman et al., 2004).

Positive symptoms and negative symptoms were assessed using the Scale for the Assessment of Positive Symptoms (SAPS; Andreasen, 1984) and the Scale for the Assessment of Negative Symptoms (SANS; Andreasen, 1983) respectively.

Functioning was scored using the Social and Occupational Functioning Assessment Scale (SOFAS; Goldman et al., 1992). Depressive symptoms were assessed using the ‘depression’ item of the Brief Psychiatric Rating Scale (BPRS; Ventura et al., 1993), which was dichotomized (moderately-to-extremely severe versus no-to-moderate symptoms). This BPRS item, and not the CDSS total, was used as a predictor, because the suicide CDSS item was already being used as the primary outcome. Also, this BPRS item’s anchors do not integrate STBs into their scoring.

For prior STBs, data was collected via chart audit, as history of STBs prior to treatment was systematically assessed and noted in the initial clinical assessment. When needed, this was complemented by CORS data. Following the Columbia-Suicide Severity Rating scale (Posner et al., 2011), all passive and active ideation were recorded as suicidal ideation, and potential self-injurious behaviors with at least some intent to die were recorded as suicide attempts.

### Statistical analyses

Analyses were performed in R Studio (V.4.1.3) and Stata (V.15.1). Chi-square and ANOVA analyses were conducted to compare baseline characteristics across sites. To identify context-specific factors associated with STBs, analyses were conducted by site. Due to the excess zeros of the dependent variable, a hurdle model (Mullahy, 1986) was conducted. This two-part model produced a better fit between predicted and observed data compared to classical regression models. First, a binomial generalized linear model was performed to examine factors associated with any baseline STBs (score 0 vs. scores > 1). Second, among those who reported STBs, an ordered logistic regression was conducted to assess factors associated with baseline severity of STBs (scores > 1). This procedure was first performed at a univariate level. Variables with a *p*-value < .10 in any model for any site were included in the multivariate models of both sites. In the multivariate ordered logistic regression, the predicted probabilities of having each CDSS suicide item score were calculated for persons presenting (1) all factors set as the reference category or the group average, (2) each significant factor individually, (3) all factors significantly associated with baseline STB severity.

Finally, a mixed-effects ordered logistic regression (Rabe-Hesketh & Skrondal, 2022), including the same covariates as the multivariate models, was performed to assess differences between sites in the 2-year STB evolution.

## Results

Of consecutive patients approached, 165 patients in Montreal and 168 in Chennai consented to the study (35 and 6 refused, respectively).

Some sample characteristics were significantly different across sites at baseline (Table 1; S. N. Iyer et al., 2022; Malla et al., 2020). In Chennai, patients were older; there was more women and individuals with partners. Baseline positive symptoms and depressive symptoms were higher in Montreal.

**Table 1.** Baseline demographic and clinical data in both sites.

Variable	Sample (*n* = 333)		Chennai (*n* = 168)		Montreal^a^ (*n* = 165)		Statistical test	*p*-Value
	*n* (%)/m (*SD*)		*n* (%)/*m* (*SD*)		*n* (%)/*m* (*SD*)			
Age at entry	25.41	(5.4)	26.6	(5.2)	24.2	(5.3)	*F* = 17.25	<.001
Gender							χ^2^ = 11.64	.002
Men	193	(58)	82	(48.8)	111	(67.3)		
Women	140	(42)	86	(51.2)	54	(32.7)		
Education (years)	12	(3.34)	11.8	(3.9)	12.2	(2.6)	*F* = 1.77	.185
Occupation status (last 4 weeks)							χ^2^ = 44.51	<.001
Student	47	(14.6)	25	(15.1)	22	(14.2)		
Employed	62	(19.3)	25	(15.1)	37	(23.9)		
Homemaker	40	(12.5)	40	(24.1)	0	(0)		
Unemployed	172	(53.6)	76	(45.8)	96	(61.9)		
Relationship status								
Single	257	(77.4)	106	(63.1)	151	(92.1)	χ^2^ = 38.85	<.001
Has a partner (married/common law/relationship)	75	(22.6)	62	(36.9)	13	(7.9)		
Living situation							χ^2^ = 23.28	<.001
Alone	18	(5.9)	2	(1.4)	16	(10.0)		
With family	265	(86.9)	140	(96.6)	125	(78.1)		
With friend	18	(5.9)	2	(1.4)	16	(10.0)		
In residence or group home	3	(1)	1	(0.7)	2	(1.3)		
Homeless	1	(0.3)	0	(0)	1	(0.6)		
Primary diagnosis (DSM-IV)^b^							χ^2^ = 26.29	<.001
Schizophrenia-spectrum disorders	259	(79.0)	150	(90.4)	109	(67.3)		
Affective psychosis	69	(21.0)	16	(9.6)	53	(32.7)		
Duration of untreated psychosis (weeks)	36.7	(75.7)	32.8	(61.1)	40.8	(88.5)	*F* = 0.87	.352
SAPS total^c^	26.9	(14.6)	19.9	(9.9)	34.6	(15)	*F* = 106.33	<.001
SANS total^d^	22.2	(14.2)	21.6	(15.7)	22.7	(12.6)	*F* = 0.45	.505
SOFAS^e^	38.9	(11.9)	38.9	(11.2)	38.9	(12.6)	*F* = 0.003	.959
Depression (BPRS item 3 – two categories)^f^							χ^2^ = 15.27	<.001
No, mild or moderate depressive symptoms with no significant impact on functioning (score < 5)	252	(76.8)	144	(85.7)	108	(67.5)		
Moderately severe, severe and extremely severe depressive symptoms with disruption in some, many or most areas of functioning (score ⩾ 5)	76	(23.2)	24	(14.3)	52	(32.5)		
History of suicidal ideation or suicide attempts prior entry							χ^2^ = 22.44	<.001
No past suicidal ideation or suicide attempts	223	(67.2)	133	(79.2)	90	(54.9)		
Past suicidal ideation	60	(18.1)	18	(10.7)	42	(25.6)		
Past suicide attempts	49	(14.8)	17	(10.1)	32	(19.5)		
Suicidal thoughts and behaviors at baseline (CDSS^g^ item 8)							χ^2^ = 11.5	.009
Absent	229	(69.8)	131	(78)	98	(61.3)		
Mild – Frequent thoughts of being better off dead, or occasional thoughts of suicide	56	(17.1)	20	(11.9)	36	(22.5)		
Moderate – Deliberately considered suicide with a plan, but no attempt	25	(7.6)	11	(6.5)	14	(8.8)		
Severe – Suicidal attempt apparently designed to end in death	18	(5.5)	6	(3.6)	12	(7.5)		

*p* <.05 is significant.

a 147 (89.1%) patients came from the larger service in the southwest of Montreal, serving a defined catchment (population of 300,000), and 18 (10.9%) patients came from the smaller service serving central Montreal (no defined catchment).

b Schizophrenia-spectrum disorders include schizophrenia, schizoaffective disorder, schizophreniform disorder, psychosis not otherwise specified, delusional disorder, brief psychotic disorder/ Affective psychosis includes Bipolar I with psychotic features and major depression with psychotic features.

c Scale for the Assessment of Positive Symptoms – higher score indicates greater severity (0–150).

d Scale for the Assessment of Negative Symptoms– higher score indicates greater severity (0–80). Items of ‘inappropriate affect’, ‘poverty of content of speech’, and the ‘attention’ subscale were removed from the SANS total since they were found unrelated to the negative symptoms domain (Malla et al., 1993).

e The Social and Occupational Functioning Assessment Scale – higher score indicates greater functioning (0–100).

f Brief Psychiatric Rating Scale.

g Calgary Depression Scale for Schizophrenia.

### STBs prior to entry and at baseline

Prior to entry into EIS, a significantly larger proportion of Montreal patients reported suicide ideation (Montreal = 25.6%, Chennai = 10.7%) or attempts (Montreal = 19.5%, Chennai = 10.1%; *p* < .001).

At baseline, more Montreal patients reported suicide ideation (Montreal = 22.5%, Chennai = 11.9%), plans (Montreal = 8.8%, Chennai = 6.5%), and attempts (Montreal = 7.5%, Chennai = 3.6%; *p* = .009).

### Factors associated with baseline STBs in Chennai

The binomial model (Table 2) comparing persons without STBs (*n* = 131) versus any STBs (*n* = 37) at baseline, shows that, adjusting for other factors, persons with severe baseline depressive symptoms had 8 times higher odds of presenting any type of STBs (*p* < .001).

**Table 2.** Factors associated with baseline suicidal thoughts and behaviors in Chennai, India.

Variable	Univariate analyses			Multivariate model (*n* = 168)		
Binomial model – No STBs^a^ (score 0) vs. any STBs (score ⩾ 1)						
	OR^b^	95% CI	*p*-Value	OR	95% CI	*p*-Value
Age at entry	1.03	[0.96, 1.11]	.414	1.00	[0.91, 1.10]	.951
Gender						
Men	R^c^			R		
Women	1.77	[0.85, 3.82]	.133	1.08	[0.43, 2.72]	.863
Relationship status						
No partner	R			R		
Has a partner	1.87	[0.89, 3.94]	.096	1.22	[0.42, 3.55]	.717
Duration of untreated psychosis (weeks)	0.99	[0.98–, 1.00]	.185			
SAPS^d^ total	0.98	[0.94, 1.02]	.306			
SANS^e^ total	1.00	[0.98, 1.03]	.780			
SOFAS^f^	0.99	[0.96, 1.02]	.600	1.01	[0.97, 1.05]	.709
Depression (BPRS^g^ item 3)						
No, mild or moderate depressive symptoms with no significant impact on functioning (score < 5)	R			R		
Moderately severe, severe and extremely severe depressive symptoms with disruption in some, many or most areas of functioning (score ⩾ 5)	9.24	[3.67, 24.60]	<.001	8.15	[3.06, 22.85]	<.001
History of suicidal ideation or suicide attempts prior entry						
No past suicidal ideation or suicide attempts	R			R		
Past suicidal ideation	2.27	[0.73, 6.49]	.135	1.80	[0.50, 5.90]	.344
Past suicide attempts	3.18	[1.06, 9.15]	.033	2.42	[0.67, 8.11]	.159
Count model – STBs severity (scores ⩾ 1) (*n* = 37)						
	Univariate analyses			Multivariate model		
	OR^b^	95% CI	*p*-value	OR	95% CI	*p*-value
Age at entry	0.96	[0.86, 1.06]	.399	0.83	[0.68, 0.98]	.054
Gender						
Men	R			R		
Women	2.73	[0.64, 11.54]	.166	0.84	[0.14, 5.10]	.845
Relationship status						
No partner	R			R		
Has a partner	3.16	[0.82, 12.14]	.091	17.17	[1.69, 303.70]	.037
Duration of untreated psychosis (weeks)	0.99	[0.96, 1.02]	.515			
SAPS^d^ total	1.03	[0.96, 1.11]	.428			
SANS^e^ total	1.01	[0.96, 1.07]	.674			
SOFAS^f^	0.94	[0.87, 1.01]	.097	0.93	[0.84, 1.02]	.153
Depression (BPRS^g^ item 3)						
No, mild or moderate depressive symptoms with no significant impact on functioning (score < 5)	R			R		
Moderately severe, severe or extremely severe depressive symptoms with disruption in some, many or most areas of functioning (score ⩾ 5)	1.41	[0.38, 5.32]	.599	0.57	[0.09, 2.96]	.521
History of suicidal ideation or suicide attempts prior entry						
No past suicidal ideation or suicide attempts	R			R		
Past suicidal ideation	2.81	[0.43, 18.20]	.274	2.37	[0.30, 19.71]	.413
Past suicide attempts	36.09	[5.46, 369.86]	.002	22.75	[2.80, 278.45]	.011

*p* <.05 is significant.

a Suicidal thoughts and behaviors.

b A score smaller than zero is a negative association while a score greater than 0 is a positive association.

c R indicates reference category.

d Scale for the Assessment of Positive Symptoms – higher score indicates greater severity (0–150).

e Scale for the Assessment of Negative Symptoms– higher score indicates greater severity (0–80).

f The Social and Occupational Functioning Assessment Scale – higher score indicates greater impairment (0–100).

g Brief Psychiatric Rating Scale.

Accounting for other factors, the count model (Table 2) including patients who reported any baseline STBs (scores > 1, *n* = 37) revealed that, those with a partner were about 17 times (*p* = .037) more likely to report suicide plans or attempt, and those with a history of suicide attempts were 23 times (*p* = .011) more likely to report suicide plans or attempts. The interaction between gender and relationship status was non-significant.

In terms of predicted probabilities, patients with both a partner *and* a history of suicide attempts who reported any baseline STBs had a predicted probability of presenting suicide ideation of 1.5%, plans of 17.2%, and attempt of 81.3% (Table S1).

### Factors associated with baseline STBs in Montreal

Accounting for other factors, the binomial model (Table 3), comparing FEP patients without STBs (*n* = 96) versus any STBs (*n* = 62), revealed that greater severity of baseline depressive symptoms and history of suicide ideation were each individually associated with about sixfold increased risk of presenting any type of STBs at baseline (*p* < .001). History of suicide attempts was associated with about 17 times greater risk of presenting any STBs (*p* < .001).

**Table 3.** Factors associated with baseline suicidal thoughts and behaviors in Montreal, Canada.

Variable	Univariate analyses			Multivariate model (*n* = 158)^a^		
Binomial model – No STBs^b^ (score 0) vs. any STBs (score ⩾ 1)						
	OR^c^	95% CI	*p*-Value	OR	95% CI	*p*-Value
Age at entry	0.94	[0.88, 1.00]	.074	0.93	[0.85, 1.01]	.094
Gender						
Men	R^d^			R		
Women	1.19	[0.60, 2.33]	.614	1.08	[0.43, 2.66]	.869
Relationship status						
No partner	R			R		
Has partner	0.98	[0.28, 3.07]	.967	1.28	[0.21, 7.02]	.782
Duration of untreated psychosis (weeks)	1.00	[1.00, 1.01]	.346			
SAPS^e^ total	1.01	[0.99, 1.04]	.251			
SANS^f^ total	1.00	[0.97, 1.02]	.811			
SOFAS^g^	0.99	[0.97, 1.02]	.615	0.99	[0.95, 1.02]	.439
Depression (BPRS^h^ item 3)						
No, mild or moderate depressive symptoms with no significant impact on functioning (score < 5)	R			R		
Moderately severe, severe or extremely severe depressive symptoms with disruption in some, many or most areas of functioning (score ⩾ 5)	7.10	[3.46, 15.16]	<.001	6.07	[2.53, 15.48]	<.001
History of suicidal ideation or suicide attempts prior entry						
No past suicidal ideation or suicide attempts	R			R		
Past suicidal ideation	6.38	[2.80, 15.17]	<.001	6.19	[2.43, 16.69]	<.001
Past suicide attempts	20.56	[7.62, 63.70]	<.001	17.08	[5.64, 59.68]	<.001
Count model – STBs severity (scores ⩾ 1) (*n* = 62)						
	Univariate analyses			Multivariate model		
	OR^c^	95% CI	*p*-Value	OR	95% CI	*p*-Value
Age at entry	0.99	[0.89, 1.10]	.844	1.06	[0.94, 1.21]	.354
Gender						
Men	R			R		
Women	2.85	[1.00, 8.18]	.051	3.61	[1.11, 12.34]	.040
Relationship status						
No partner	R			R		
Has partner	0.29	[0.03, 2.80]	.278	0.12	[0.00, 1.12]	.099
Duration of untreated psychosis (weeks)	1.00	[1.00, 1.01]	.438			
SAPS^e^ total	1.02	[0.99, 1.05]	.274			
SANS^f^ total	1.01	[0.97, 1.05]	.743			
SOFAS^g^	1.00	[0.97, 1.04]	.840	0.99	[0.94, 1.04]	.684
Depression (BPRS^h^ item 3)						
No, mild or moderate depressive symptoms with no significant impact (score < 5)	R			R		
Moderately severe, severe and extremely severe depressive symptoms with disruption in some, many or most areas of functioning (score ⩾ 5)	1.85	[0.66, 5.16]	.238	2.24	[0.73, 7.28]	.173
History of suicidal ideation or suicide attempts prior entry						
No past suicidal ideation or suicide attempts	R			R		
Past suicidal ideation	1.49	[0.33, 8.14]	.618	1.50	[0.30, 9.03]	.633
Past suicide attempts	8.41	[2.11, 43.68]	.007	8.54	[1.94, 49.37]	.010

*p* <.05 is significant.

a Seven patients in Montreal were excluded because of missing data on selected factors.

b Suicidal thoughts and behaviors.

c A score smaller than zero is a negative association while a score greater than 0 is a positive association.

d R indicates reference category.

e Scale for the Assessment of Positive Symptoms – higher score indicates greater severity (0–150).

f Scale for the Assessment of Negative Symptoms– higher score indicates greater severity (0–80).

g The Social and Occupational Functioning Assessment Scale – higher score indicates greater impairment (0–100).

h Brief Psychiatric Rating Scale.

The count model (Table 3) shows that, among persons who reported any baseline STBs (scores > 1, *n* = 62), women had a 3.6-fold (*p* = .04) increased risk of reporting suicide plans or attempt, and those with a history of suicide attempts had an 8.5-fold higher risk of suicide plans or attempt (*p* = .01).

In terms of predicted probabilities, among women who reported past suicide attempts and any baseline STBs, the predicted probabilities were 21.6% for suicide ideation, 33.7% for plans and 44.7% for attempt (Table S2).

### Evolution of STBs

#### Descriptives

In Chennai, 52 (30.1%) patients reported STBs from baseline to the end of follow-up. Of these, 47 reported suicide ideation (18 admission only, 13 admission *and* during follow-up, 16 only during follow-up) and 9 attempted suicide (7 attempted once). Seven attempts occurred in the first 2 months of follow-up. Of the 9 attempters, three women died from their suicide attempts in the first 4 months of treatment (Table S3 for details).

In Montreal, 92 (55.8%) patients reported STBs from baseline to the end of follow-up: 85 of them reported suicide ideation (21 admission only, 29 admission *and* during follow-up, 35 only during follow-up) and 15 attempted suicide (all attempted only once, 12 attempted at baseline). No deaths by suicide occurred.

At both sites, a greater proportion of persons reported STBs at baseline, with the largest decrease occurring between admission and the second month (Table S4).

#### Regression analysis

**Figure 1.**
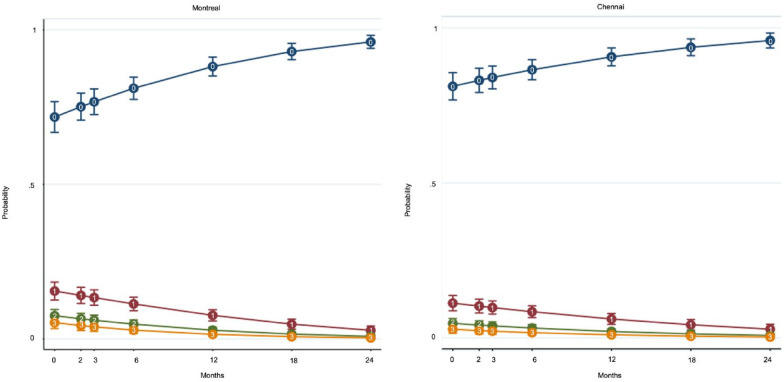
0: No suicidal thoughts or behaviors, 1: Suicidal ideation, 2: Suicidal ideation with a plan, 3: Suicide attempt.

Controlling for several demographic and clinical variables, ‘site’ was significant. As shown in Figure 1, Montreal patients had a higher risk of presenting STBs throughout follow-up. There was also a significant ‘time’ effect, that is, a decrease in STBs over the 2-year follow-up (Table 4). No time-by-site interaction was detected. Those with more severe depressive symptoms, a history of STBs or with partners were at greater risk of presenting STBs over follow-up. Although relationship-by-site interaction was not significant, more persons had partners in Chennai.

**Table 4.** Mixed-effects ordered logistic regression to compare the evolution of suicidal thoughts and behaviors by site (*n* = 326)^a^.

Variable	OR	95% CI	*p*-Value
Months	0.87	[0.84, 0.90]	.000
Site			
Montreal	R^b^		
Chennai	0.44	[0.23, 0.81]	.009
Time by site interaction			
Montreal	R		
Chennai	1.04	[0.99, 1.09]	.122
Age at entry	0.95	[0.90, 1.00]	.068
Gender			
Men	R		
Women	1.42	[0.84, 241]	.192
Relationship status			
No partner	R		
Has partner	2.23	[1.07, 4.64]	.032
SOFAS^c^	1.01	[0.98, 1.03]	.621
Depression (BPRS item 3)^d^			
No, mild or moderate depressive symptoms with no significant impact on functioning (score < 5)	R		
Moderately severe, severe or extremely severe depressive symptoms with disruption in some, many or most areas of functioning (score ⩾ 5)	4.42	[2.50, 7.79]	.000
History of suicidal ideation or suicide attempts prior entry			
No past suicidal ideation or suicide attempts	R		
Past suicidal ideation	2.96	[1.57, 5.59]	.001
Past suicide attempts	5.43	[2.76, 10.67]	.000

*p* <.05 is significant.

a Seven patients in Montreal were excluded because of missing data on selected predictors.

b R indicates reference category.

c The Social and Occupational Functioning Assessment Scale – higher score indicates greater impairment (0–100).

d Brief Psychiatric Rating Scale.

## Discussion

This is the first study to compare STBs in two FEP cohorts between an HIC (Montreal, Canada) and an LMIC (Chennai, India). At both sites, STB rates were highest at admission, and then decreased, with the largest decline in the first 2 months, suggesting that early access to specialized EIS for psychosis tailored to youth needs and stage of illness may contribute to reducing STBs across contexts by providing hope and support. This is consistent with previous studies (abeit all in HICs; Canal-Rivero et al., 2016; Chen et al., 2011; Fedyszyn et al., 2010; Harris et al., 2008; Moe et al., 2022; Sicotte et al., 2021, 2023).

A greater proportion of persons reported STBs prior to entry into EIS, at admission and throughout follow-up in Montreal. Our work extends for the first time to FEP the findings that STB rates in psychosis are higher in HICs compared to LMICs (Bai et al., 2021; Lu et al., 2020). Further, in our study, rates of STBs in Montreal were consistent with those reported in FEP in HICs, whereas rates in Chennai were generally lower (Sicotte et al., 2021) (Table S5).

While rates of STBs are usually higher in young adults in the general population in LMICs compared to the general population in HICs (Robinson & Krysinska, 2019; Uddin et al., 2019), the opposite seems to be the case in FEP (at least for Chennai). Although suicide ideation and attempts were higher in Montreal, three individuals, all women, died by suicide in Chennai early in the follow-up, whereas no suicide occurred in Montreal. Despite the low number of suicide, this specific finding is consistent with the general population as suicide death rate for youth aged 15 to 29 years is much higher in India (25.5/100,000) (Patel et al., 2012) than in Canada (11.9/100,000) (Statistique Canada, 2020). Chennai has one of the highest suicide rates among Indian megacities (National Crime Records Bureau, 2022), and in India, young women are the groups most at risk for suicide, owing to factors like arranged and early marriage, low social status, low rights, and depressive symptoms (Dandona et al., 2018).

### Why were rates of STBs different across the two sites?

Different hypotheses may explain our finding of lower STB rates in Chennai. One possible explanation is that STBs are underreported because of the strong stigma of suicide and psychosis in India (Gupta & Basera, 2023; Mascayano et al., 2015). Suicide has only recently been decriminalized, and Indian youth have more negative attitudes toward suicide than their HIC counterparts and feel that society’s judgment of suicide affects and shames the family (Colucci & Lester, 2020; Helal Mehtab et al., 2022). Individuals with schizophrenia, especially women, also report high rates of perceived community and family stigma (Mascayano et al., 2015). Reporting STBs may be particularly difficult for FEP patients who are coping with illness onset and may already fear discrimination. Patients may also be less willing to disclose STBs out of fear of judgment by the clinical team or disappointment of their relatives if they were informed. While relevant across contexts, social-interpersonal deterrents to reporting STBs may be stronger in India.

Another hypothesis is that STBs are lower in Chennai due partly to lower expectations from (and therefore pressure on) persons with mental illnesses in India, as previously reported with the same samples (S. N. Iyer et al., 2023). In the general population in India, however, expectations from young people and associated pressures are alarmingly high (Mitra & Arnett, 2021; Seiter & Nelson, 2011). This may explain why we found lower STBs in the Chennai FEP cohort although in the general population, STB rates in youth are higher in India than in HICs (Robinson & Krysinska, 2019; Uddin et al., 2019).

STBs were strongly associated with depressive symptoms in both cohorts. The lower number of patients in Chennai with severe depressive symptoms could have contributed to their lower STBs rate. At both sites, while depressive symptoms increased risk of presenting any STBs, depressive symptoms were not associated with greater severity of baseline STBs, suggesting that other factors explain transition from suicidal thoughts to plans or attempts, and possibly suicide death. As such, history of prior suicide attempts was associated with greater risk of suicide plans and attempts at baseline in both cohorts.

History of prior suicidal ideation was however associated with baseline STBs only in Montreal. In HICs, suicidal behaviors are often preceded by suicidal ideation, whereas in some LMICs, some suicidal behaviors may be more impulsive and likely to occur after a difficult life event with no prior ideation (Robinson & Krysinska, 2019; Uddin et al., 2019). However, all three women who died in Chennai reported suicidal ideation/attempts before entry and at baseline.

The course of psychosis has been reported to be less favorable for several outcomes (e.g. symptoms, disengagement, recovery) in HICs compared to some LMICs (particularly India), including in our cohort (S. N. Iyer et al., 2022; Malla et al., 2020). This may have contributed to greater risk of STBs during follow-up among Montreal patients, since STBs later in follow-up may be related to psychosis sequelae such as hopelessness, social isolation, and lower quality of life (Ayesa-Arriola et al., 2015).

Finally, health system context differences may also have played a role. In Montreal, the emergency services within the EIS’ larger premises and the physicians being more oriented toward risk management (MacDonald et al., 2023), as well as the higher uptake of legal means to hospitalize patients against their will (or the family’s) in case of suicidal risk, may have contributed to lower risk of death by suicide. For example, in one suicide death in Chennai, the patient’s husband refused the immediate admission advised by the treating team, and the suicide occurred few hours later.

### The influence of relationship status and gender is shaped by context

In Chennai only, being in a relationship was associated with higher risk for severe STBs. Those with both a partner *and* history of suicide attempts prior to entry, who reported baseline STBs in Chennai had a predicted probability of attempting suicide of 82%. More women in the Chennai sample were married. Two of the three women deceased by suicide were married and two had a history of suicide attempts; these factors may be important indicators of suicide risk as in the Indian general population (Dandona et al., 2018; Rane & Nadkarni, 2014). Married young women in India have increased risk of suicide, due, in part, to domestic violence, economic dependence, young motherhood, dowry disputes, etc. (Dandona et al., 2018; Rane & Nadkarni, 2014). Furthermore, married people with psychosis, especially women, often face greater stigma and shunning in India (Thara et al., 2003; Thara & Srinivasan, 1997a, 1997b).

Gender was not statistically associated with STBs in Chennai (but the three suicide decedents were women). In Montreal, women were more likely to engage in suicide plans and attempts which is consistent with the general population of HICs, where more women report STBs, but men have higher suicide rates, which may be partly explained by their greater use of lethal means (WHO, 2014). The means used (pesticides intoxication, hanging, self-immolation) by the three women who died are among the most common suicide means used by Indian women (Aggarwal, 2015).

### Strengths and limitations

Our study has several strengths, including the comparison of similarly defined, well-characterized FEP patients from two similar EIS across an HIC and an LMIC. The seven measures of STBs allowed for a clear capture of the 2-year evolution. Suicide deaths were documented at both sites, with the additional source of coroners’ reports for those lost to follow-up in Montreal (no patients were lost to follow-up in Chennai). Using a hurdle model allowed us to account for excess zeros, and go well beyond merely comparing the absence or presence of STBs to examine factors associated with STB severity (Bethell et al., 2010). Conducting the analyses by site allowed the identification of site-specific factors.

Our study has some limitations, such as the sample size per site. But, the very low number of missing data and of patients who declined consent strengthens our samples’ representativeness of all young persons with FEP at both sites. We cannot exclude that some differences between cohorts prevalence could be due to sampling biases (e.g. all cases in a sectorized health system with a defined catchment area (Montreal) versus those seeking this specific help in a less geographically bounded area (Chennai)). However, we controlled for pertinent variables to mitigate this bias. Our assessment of STBs and depressive symptoms were both drawn from one item of standardized scales, which may have hindered a more nuanced assessment of these constructs. Factors potentially associated with STBs were examined only at admission. Despite our efforts to distinguish between suicidal ideation, plans and attempts, all STBs were sometimes combined in our statistical analyses, as in the longitudinal model, potentially hindering our ability to identify the influence of certain factors on specific STB types. Given that socio-cultural and politico-economic factors would be as or more important than individual factors in understanding suicide in LMICs (Gupta & Basera, 2023), future studies should consider such factors, including family relationships, religious beliefs, rights. Since our study was conducted with only one cohort in Montreal and in Chennai, our findings cannot be overly generalized to other regions. Our study does however strengthen the urgent case for more research on STBs and suicides in psychosis in LMICs, a striking gap currently (Thara & Srinivasan, 1997a).

## Conclusion

Across geo-cultural contexts, the period around treatment onset seems crucial and represents a critical window for suicide prevention, with STBs decreasing for most patients at both sites in the first 2 months after treatment initiation. Nonetheless, in both cohorts, a minority of patients presented STBs for the first time and others multiple times during follow-up. Thus, suicide assessment, considering factors that may operate across contexts (depressive symptoms, prior suicide attempts) and those that may be context-specific (gender, marital status, prior suicide ideation), should be conducted upon entry and throughout follow-up.

## Supplemental Material

sj-docx-1-isp-10.1177_00207640231214979 – Supplemental material for Similar and different? A cross-cultural comparison of the prevalence, course of and factors associated with suicidal thoughts and behaviors in first-episode psychosis in Chennai, India and Montreal, Canada Supplemental material, sj-docx-1-isp-10.1177_00207640231214979 for Similar and different? A cross-cultural comparison of the prevalence, course of and factors associated with suicidal thoughts and behaviors in first-episode psychosis in Chennai, India and Montreal, Canada by Roxanne Sicotte, Amal Abdel-Baki, Greeshma Mohan, Daniel Rabouin, Ashok Malla, Ramachandran Padmavati, Laura Moro, Ridha Joober, Thara Rangaswamy and Srividya N. Iyer in International Journal of Social Psychiatry
