# Supplementary material for: Similar and different? A cross-cultural comparison of the prevalence, course of and factors associated with suicidal thoughts and behaviors in first-episode psychosis in Chennai, India and Montreal, Canada
Source: Int J Soc Psychiatry. 2024 Jan 4;70(3):457–69. doi: 10.1177/00207640231214979 (PMC11067410; doi:10.1177/00207640231214979)
Supplement: sj-docx-1-isp-10.1177_00207640231214979 – Supplemental material for Similar and different? A cross-cultural comparison of the prevalence, course of and factors associated with suicidal thoughts and behaviors in first-episode psychosis in Chennai, India and Montreal, Canada [file sj-docx-1-isp-10.1177_00207640231214979.docx]

**Supplementary material**

**Similar and Different? A cross-cultural comparison of the prevalence, course of and factors associated with suicidal thoughts and behaviors in first-episode psychosis** **in Chennai, India and Montreal, Canada**

Sicotte R., Abdel-Baki A., Mohan G., Rabouin D., Malla A., Padmavati R., Moro L., Joober, R., Rangaswamy T., Iyer SN*

**Corresponding author:** Srividya N. Iyer ([srividya.iyer@mcgill.ca](mailto:srividya.iyer@mcgill.ca)), Prevention and Early Intervention Program for Psychosis (PEPP-Montreal), Douglas Mental Health University Institute, Montreal, Canada

| Supplementary Table 1 | Predicted probabilities of severity of STBs among patients who reported STBs at baseline (scores ≥ 1) in Chennai, India (n=37) | P. 2 |
| --- | --- | --- |
| Supplementary Table 2 | Predicted probabilities of severity of STBs among patients who reported STBs at baseline (scores ≥ 1) in Montreal, Canada (n=62) | P. 3 |
| Supplementary Table 3 | Baseline demographic and clinical data of persons who died by suicide | P. 4 |
| Supplementary Table 4 | Frequency of suicidal thoughts and behaviors | P. 5 |
| Supplementary Table 5 | Rates of suicidal thoughts and behaviors compared to those reported in the literature in first-episode psychosis | P. 6 |

**Table S1. Predicted probabilities of severity of STBs^a^ among patients who reported STBs at baseline (scores ≥ 1) in Chennai, India (n=37)**

| **Gender** | **Age at entry** | **SOFAS** | **Relationship status** | **Depression  (BPRS^b^ item 3)** | **History of suicidal ideation or suicide attempts** | **Predicted probabilities** | | |
| --- | --- | --- | --- | --- | --- | --- | --- | --- |
|  |  |  |  |  |  | **Suicidal ideation** | **Suicide planning** | **Suicide attempt** |
| Men | 26.6 | 38.9 | No partner | no, mild or moderate depressive symptoms with no significant impact on functioning (score < 5) | No past suicidal ideation or attempts | 84.5% | 14.3% | 1.2% |
| Men | 26.6 | 38.9 | **Has a partner** | no, mild or moderate depressive symptoms with no significant impact on functioning (score < 5) | No past suicidal ideation or attempts | 24.2% | 59.1% | 16.7% |
| Men | 26.6 | 38.9 | No partner | no, mild or moderate depressive symptoms with no significant impact on functioning (score < 5) | **Past suicide attempts** | 19.4% | 59.6% | 21% |
| Men | 26.6 | 38.9 | **Has a partner** | no, mild or moderate depressive symptoms with no significant impact on functioning (score < 5) | **Past suicide attempt** | 1.4% | 16.6% | 82% |
| Men | **21.4** | 38.9 | **Has a partner** | no, mild or moderate depressive symptoms with no significant impact on functioning (score < 5) | **Past suicide attempt** | 0.5% | 7.3% | 92.2% |
| Men | **31.8** | 38.9 | **Has a partner** | no, mild or moderate depressive symptoms with no significant impact on functioning (score < 5) | **Past suicide attempt** | 3.5% | 32.6% | 63.9% |

Among persons who reported STBs at baseline (score ≥ 1, n=37), the predicted probabilities of having each score of the dependent variable (suicide item of the CDSS) are calculated for each of the significant factors in the count model.

The other categorical variables of the model are set as the reference category and the continuous variables are set as the group mean.

^a^ Suicidal thoughts and behaviors

^b^ Brief Psychiatric Rating Scale

**Table S2. Predicted probabilities of severity of STBs^a^ among patients who reported STBs at baseline (scores ≥ 1) in Montreal, Canada (n=62)**

| **Gender** | **Age at entry** | **SOFAS** | **Relationship status** | **Depression  (BPRS^b^ item 3)** | **History of suicidal ideation or suicide attempts** | **Predicted probabilities** | | |
| --- | --- | --- | --- | --- | --- | --- | --- | --- |
|  |  |  |  |  |  | **Suicidal ideation** | **Suicide planning** | **Suicide attempt** |
| Men | 24.2 | 38.9 | No partner | no, mild or moderate depressive symptoms with no significant impact on functioning (score < 5) | No past suicidal ideation or attempts | 89.1% | 8.3% | 2.6% |
| **Women** | 24.2 | 38.9 | No partner | no, mild or moderate depressive symptoms with no significant impact on functioning (score < 5) | No past suicidal ideation or attempts | 69.3% | 21.7% | 9% |
| Men | 24.2 | 38.9 | No partner | no, mild or moderate depressive symptoms with no significant impact on functioning (score < 5) | **Past suicide attempts** | 48.9% | 32.2% | 18.9% |
| **Women** | 24.2 | 38.9 | No partner | no, mild or moderate depressive symptoms with no significant impact on functioning (score < 5) | **Past suicide attempt** | 20.9% | 33.4% | 45.7% |

Among persons who reported STBs at baseline (score ≥ 1, n=62), the predicted probabilities of having each score of the dependent variable (suicide item of the CDSS) are calculated for each of the significant factors in the count model.

The other categorical variables of the model are set as the reference category and the continuous variables are set as the group mean.

^a^ Suicidal thoughts and behaviors

^b^ Brief Psychiatric Rating Scale

**Table S3. Baseline demographic and clinical data of persons who died by suicide**

| **Variable** | **Persons who died by suicide (n=3)** n (%)/  m (sd) | |
| --- | --- | --- |
| **Site (Chennai)** | 3 |  |
| **Age at entry** | 31 | (3.6) |
| **Gender** |  |  |
| Women | 3 |  |
| **Education (years)** | 11 | (5.3) |
| **Occupation status (last 4 weeks)** |  |  |
| Homemaker | 2 |  |
| Unemployed | 1 |  |
| **Relationship status** |  |  |
| Single | 1 |  |
| Married | 2 |  |
| **Living situation** |  |  |
| With family | 3 |  |
| **Primary diagnosis (DSM-IV)** |  |  |
| Schizophrenia-spectrum disorders | 2 |  |
| Affective psychosis | 1 |  |
| **Substance abuse & dependence diagnosis** | 0 |  |
| **SAPS total**^a^ | 25.7 | (5.5) |
| **SANS total**^b^ | 32 | (13.1) |
| **SOFAS**^c^ | 38.3 | (20.2) |
| **Depression (BPRS**^d^ **item 3– two categories)** |  |  |
| no, mild or moderate depressive symptoms with no significant impact on functioning (score < 5) | 2 |  |
| Moderately severe, severe and extremely severe depressive symptoms with disruption in some, many or most areas of functioning (score ≥ 5) | 1 |  |
| No past suicidal ideation or suicide attempts | . |  |
| Past suicidal ideation | 1 | (33.3) |
| Past suicide attempts | 2 | (66.7) |
| **Suicidal thoughts and behaviors at baseline (CDSS**^e^ **item 8)** |  |  |
| Absent | . |  |
| Mild – Frequent thoughts of being better off dead, or occasional thoughts of suicide | . |  |
| Moderate – Deliberately considered suicide with a plan, but no attempt | 2 | (66.7) |
| Severe – Suicidal attempt apparently designed to end in death | 1 | (33.3) |
| **Means of suicide** |  |  |
| Self-immolation | 1 |  |
| Pesticide | 1 |  |
| Hanging | 1 |  |
| **Time to suicide** |  |  |
| Month 1 | 1 |  |
| Month 2 | 1 |  |
| Month 4 | 1 |  |

^a^ Scale for the Assessment of Positive Symptoms – higher score indicates greater severity (0-150)

^b^ Scale for the Assessment of Negative Symptoms– higher score indicates greater severity (0-80)

^c^ The Social and Occupational Functioning Assessment Scale – higher score indicates greater impairment (0-100)

^d^ Brief Psychiatric Rating Scale

^e^ Calgary Depression Scale for Schizophrenia

**Table S4. Frequency of suicidal thoughts and behaviors**

| **Time point** | **Sample**  **(n=333)** | | **Chennai**  **(n=168)**^a^  n (%) | | **Montreal  (n=165)** n (%) | |
| --- | --- | --- | --- | --- | --- | --- |
| **Admission** | **328**^b^ |  | **168** |  | **160** |  |
| Absent | 229 | (68.8) | 131 | (78) | 98 | (61.3) |
| Mild – Frequent thoughts of being better off dead, or occasional thoughts of suicide | 56 | (17.1) | 20 | (11.9) | 36 | (22.5) |
| Moderate – Deliberately considered suicide with a plan, but no attempt | 25 | (7.6) | 11 | (6.5) | 14 | (8.8) |
| Severe – Suicidal attempt apparently designed to end in death | 18 | (5.5) | 6 | (3.6) | 12 | (7.5) |
| **Month 2** | **304** |  | **165** |  | **139** |  |
| Absent | 271 | (89.1) | 153 | (92.7) | 118 | (84.9) |
| Mild | 23 | (7.6) | 7 | (4.2) | 16 | (11.5) |
| Moderate | 9 | (3) | 4 | (2.4) | 5 | (3.6) |
| Severe | 1 | (0.3) | 1 | (0.6) | 0 | (0) |
| **Month 3** | **314** |  | **161** |  | **153** |  |
| Absent | 279 | (88.9) | 156 | (96.9) | 123 | (80.4) |
| Mild | 27 | (8.6) | 3 | (1.9) | 24 | (15.7) |
| Moderate | 6 | (1.9) | 2 | (1.2) | 4 | (2.6) |
| Severe | 2 | (0.6) | 0 | (0) | 2 | (1.3) |
| **Month 6** | **322** |  | **162** |  | **160** |  |
| Absent | 293 | (91) | 154 | (95.1) | 139 | (86.9) |
| Mild | 19 | (5.9) | 3 | (1.9) | 16 | (10) |
| Moderate | 10 | (3.1) | 5 | (3.1) | 5 | (3.1) |
| Severe | 0 | (0) | 0 | (0) | 0 | (0) |
| **Month 12** | **304** |  | **159** |  | **145** |  |
| Absent | 284 | (93.4) | 149 | (93.7) | 135 | (93.1) |
| Mild | 16 | (5.3) | 7 | (4.4) | 9 | (6.2) |
| Moderate | 3 | (1) | 2 | (1.3) | 1 | (0.7) |
| Severe | 1 | (0.3) | 1 | (0.6) | 0 | (0) |
| **Month 18** | **298** |  | **157** |  | **141** |  |
| Absent | 277 | (93) | 152 | (96.8) | 125 | (88.7) |
| Mild | 17 | (5.7) | 4 | (2.5) | 13 | (9.2) |
| Moderate | 2 | (0.7) | 0 | (0) | 2 | (1.4) |
| Severe | 2 | (0.7) | 1 | (0.6) | 1 | (0.7) |
| **Month 24** | **296** |  | **163** |  | **133** |  |
| Absent | 276 | (93.2) | 153 | (93.9) | 123 | (92.5) |
| Mild | 12 | (4.1) | 5 | (3.1) | 7 | (5.3) |
| Moderate | 6 | (2) | 3 | (1.8) | 3 | (2.3) |
| Severe | 2 | (0.7) | 2 | (1.2) | 0 | (0) |

^a^ The three deaths by suicide that occurred in the first four months in the Chennai cohort are not included in these suicide attempts

^b^ Sample size (n) varies at different time points due to missing data

**Table S5. Rates of suicidal thoughts and behaviors compared to those reported in the literature in first-episode psychosis**

|  | **Suicide ideation** | | | | **Suicide attempts** | | | |
| --- | --- | --- | --- | --- | --- | --- | --- | --- |
|  | **Our study** | | **Rates reported in the literature in FEP^1^** | | **Our study** | | **Rates reported in the literature in FEP^1^** | |
| **Site** | Prior entry into services | At admission | Prior entry into services | At admission | Prior entry into services | At admission | Prior entry into services | At admission |
| **Chennai** | 10.7% | 18.4% | 21.7-31.4% | 30.6-56.5% | 10.1% | 3.6% | 7.3-33% | 3.3-9.6% |
| **Montreal** | 25.6% | 31.3% |  |  | 19.5% | 7.5% |  |  |

1. Sicotte R, Iyer SN, Kiepura B et al. 2021. A systematic review of longitudinal studies of suicidal thoughts and behaviors in first-episode psychosis: Course and associated factors. *Soc Psychiatry Psychiatr Epidemiol. 56*(12):2117–2154.
